# Haemophagocytic Lymphohistiocytosis in an Elderly Patient after Recent Cardiac Surgery

**DOI:** 10.1155/2021/9232308

**Published:** 2021-01-15

**Authors:** Eoghan Burke, Patricia Harkins, Jennifer Kieran

**Affiliations:** ^1^Royal College of Surgeons in Ireland, Dublin, Ireland; ^2^St James's Hospital, Department of Medicine, Dublin, Ireland

## Abstract

**Introduction:**

The underlying pathophysiology of haemophagocytic lymphohistiocytosis (HLH) is characterised by excessive inflammation and tissue destruction secondary to abnormal immune activation. The term primary HLH refers to a genetic abnormality that predisposes to the condition whereas secondary refers to HLH being triggered by an underlying condition such as infection (often Epstein Barr Virus), autoimmune, or neoplastic disease. Its variable clinical presentation poses an obstacle to prompt diagnosis in the elderly patient.

**Case:**

A 70-year-old Caucasian man was admitted to hospital from a convalescence center with symptoms of fatigue, fever, decreased oral intake, and increasing shortness of breath on exertion. The patient was three weeks after coronary artery bypass grafting. Over the next two weeks, the patient continued to deteriorate both clinically and biochemically. The patient met criteria for haemophagocytic lymphohistiocytosis, likely driven by EBV infection. Bone marrow biopsy supported the diagnosis with evidence of active phagocytosis. The patient was commenced on high-dose dexamethasone and reviewed by haematology with further molecular testing confirming the diagnosis. *Discussion*. LH is becoming more common in older patients. We propose that new guidelines be developed to aid its prompt diagnosis in this age group.

## 1. Introduction

The underlying pathophysiology of haemophagocytic lymphohistiocytosis (HLH) is characterised by excessive inflammation and tissue destruction secondary to abnormal immune activation. This hyperimmune state is thought to be secondary to a failure of normal downregulation of macrophage activity [1]. These macrophages release large volumes of proinflammatory cytokines which are thought to be responsible for the development of multiorgan failure in these patients [2]. Broadly speaking, it can be classified into primary and secondary. The term primary HLH refers to a genetic abnormality that predisposes to the condition whereas secondary refers to HLH being triggered by an underlying condition such as infection (often Epstein Barr Virus), autoimmune, or neoplastic disease [3]. Its variable clinical presentation poses an obstacle to prompt diagnosis in the elderly patient.

## 2. Case Report

A 70-year-old Caucasian man was admitted to hospital from a convalescence center with symptoms of fatigue, fever, decreased oral intake, and increasing shortness of breath on exertion. The patient was three weeks after coronary artery bypass grafting. His postoperative course included multiple blood transfusions secondary to intraoperative losses. The patient denied any chest pain, cough, nausea, diarrhoea, night sweats, or urinary symptoms concerning for post-op infection. His past medical history was remarkable for hepatic sarcoidosis, atrial fibrillation, hypertension, hyperlipidemia, and stage 3 chronic kidney disease.

On admission, clinical examination revealed a temperature of 39.6 degrees Celsius, heart rate was 80 beats per minute, blood pressure was 122/50 mmHg, respiratory rate was 16 breaths per minute, and he was saturating at 96% on room air. Heart sounds 1 and 2 were present and normal with no added sounds, and minimal crepitations in the left lung base were noted on auscultation. The abdomen was soft and nontender with no organomegaly. The patient was alert and oriented.

Laboratory results are displayed in Table 1. Chest X-ray revealed evidence of his recent sternotomy. A septic screen was performed with blood cultures and midstream urine sample taken for culture and sensitivity. The patient was commenced on IV piperacillin and tazobactam and admitted to the ward with the initial diagnosis of pneumonia.

Over the next two weeks, the patient continued to deteriorate both clinically and biochemically. CT thorax indicated small bilateral pleural effusions and a small pericardial effusion. Transthoracic and transoesophageal echo did not indicate any evidence of haemodynamic compromise caused by the pericardial effusion. He was reviewed by the cardiothoracic team who advised conservative management.

MSU and blood cultures did not grow any pathogen. The patient developed pancytopenia and worsening LFTs (Table 1).

The patient became frankly jaundiced with scleral icterus. His levels of consciousness began to fluctuate, and he was spiking temperatures, desaturating, and becoming increasingly hypotensive. Ultrasound of his liver did not indicate any intra- or extrabiliary tract obstruction, focal liver lesion, or sonographic evidence of acute cholecystitis. CT abdomen indicated widespread lymphadenopathy which was likely reactive.

Serology for hepatitis B and C and HIV was negative. Serum plasma electrophoresis did not detect monoclonal bands. Alpha 1 antitrypsin and ceruloplasmin were within normal limits. Antinuclear, neutrophil-cytoplasmic, smooth muscle, mitochondrial, and liver-kidney antibodies were all negative. Iron overload studies indicated the following: iron within normal limits, a low total iron binding capacity, raised transferrin saturation, and ferritin of greater than 30,000 ug/L. Serum triglycerides were elevated at 3.22 mmol/L, and fibrinogen was noted to be low at 1.2 g/L. EBV DNA was detected using PCR and EBV. Nuclear antigen IgG became positive. There was no evidence of serological analysis being performed in this patient prior to surgery, nor was it evident in his medical chart from previous admissions.

**Figure 1:**
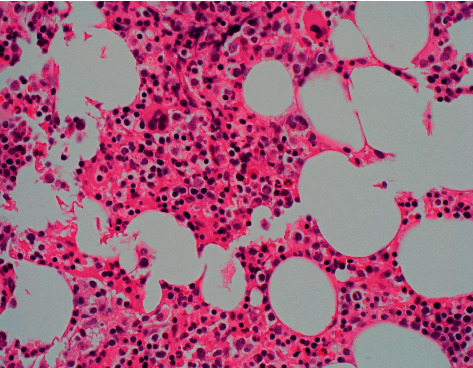
Haemotoxylin and eosin stained bone marrow trephine (x60) showing erythroid hyperplasia, normal megakaryocytes, and a subtle infiltrate of small lymphocytes and histiocytes.

The patient met criteria for haemophagocytic lymphohistiocytosis, likely driven by EBV infection. Bone marrow biopsy supported the diagnosis with evidence of active phagocytosis (Figure 1). The patient was commenced on high-dose dexamethasone and reviewed by haematology with further molecular testing confirming the diagnosis.

The patient made a full recovery and was subsequently discharged back to a convalescence center for further rehabilitation.

## 3. Discussion

HLH is a rare and devastating disorder of uncontrolled immune activation which is well described in paediatric populations but far less is known about an appropriate approach to diagnosis and treatment in older populations [4].

The incidence of HLH in the paediatric population is estimated at 1 to 225 per 300,000 live births [5]. The incidence in the adult population is unknown.

Adult patients with HLH will present with a myriad of clinical and laboratory abnormalities which often delays diagnosis. In an attempt to streamline early diagnosis, the Histiocyte Society has developed criteria for the diagnosis of HLH (see Table 2) [6].

It is important to understand, however, that these diagnostic criteria were developed to aid the diagnosis of HLH in the paediatric population and may be less effective in adults [7]. A serum ferritin level greater than 10,000 ng/ml, for example, is approximately 95% sensitive and specific for HLH in the paediatric population [8]; however, in the adult population, the differential for a similar serum ferritin level is much broader and includes more common pathologies such as renal and liver failure, sepsis, and haemochromatosis [9].

The mechanism of EBV infection in our patient is not clear. One potential mechanism is via blood transfusions the patient received intra- and postoperatively following his cardiac surgery. This has been well documented predominantly in the paediatric population [10].

The current treatment paradigms for HLH in the adult population were developed from studies in the paediatric population, namely, the HLH-94 study which was a large prospective paediatric study conducted in patients less than 16 years old [11].

In light of the increasing cases of HLH reported in adult populations, we propose that new guidelines be developed to aid its prompt diagnosis in this age group. Similarly, adult specific treatment guidelines must be developed in order to combat this frequently fatal pathology.

## Figures and Tables

**Table 1:** Laboratory data.

Indices	Day 1: emergency department	Day 5	Day 13	Others	Reference range adults
Haemoglobin (g/dl)	9.0		8.7		13.5 – 18.0
Mean corpuscular volume	82.8		79.4		83.0 – 99.0
Reticulocyte count (%)		1.1%			0.4 – 1.9
Nucleated red blood cell (*∗* 10^9^/L)		0.0	0.1		
White cell count (*∗* 10^9^/L)	4.1		2.7		4.0 – 11.0
Neutrophils (*∗* 10^9^/L)	3.1		1.5		2.0 – 7.5
Platelet count (*∗* 10^9^/L)	227		45		140 – 450
Sodium (mmol/L)	126		134		136 – 145
Potassium (mmol/L)	4.4		4.0		3.5 – 5.3
Urea (mmol/L)	9.4		8.8		2.5 – 7.8
Creatinine (*μ*mol/L)	180		132		59 – 104
Total protein (g/L)	67		57		66 – 87
Serum albumin (g/L)	30		19		33 – 50
Alkaline phosphatase (IU/L)	353		1462		40 – 129
Gamma-glutamyl transferase (IU/L)	403		635		10 – 71
Bilirubin (*μ*mol/L)	14		271		0 – 21
Alanine aminotransferase (IU/L)	148		336		0 – 41
Aspartate aminotransferase (IU/L)	134		1013		0 – 40
Lactic dehydrogenase (IU/L)	262				135 – 250
Corrected calcium (mmol/L)	2.10		2.13		2.15 – 2.50
Magnesium (mmol/L)	0.86		0.90		0.70 – 1.00
Phosphate (mmol/L)	1.12		1.00		0.81 – 1.45
C-reactive protein (mg/L)	80.46		16.49		0.00 – 5.00
Serum angiotension-converting enzyme (ACE) (IU/L)				106	<8 – 65
IgG (g/L)				16.35	6.26 – 14.96
IgM (g/L)				3.96	0.47 – 1.82
IgA (g/L)				14.52	0.62 – 2.90
Alpha 1 antitrypsin (g/L)				2.15	1.10 – 2.20
Ceruloplasmin (g/L)				0.35	0.22 – 0.66
Iron (µmol/L)				18	14 – 31
Unsaturated iron binding capacity (*μ*mol/L)				10	20 – 66
Transferrin saturations (%)				64	30 – 40
Ferritin (ug/L)				35 164.8	23.0 – 393.0
Fibrinogen (g/L)				1.2	1.9 – 3.5
Haptoglobins (g/L)				<0.24	0.45 – 2.05
Triglycerides (mmol/L)				3.22	0.50 – 1.70
EBV DNA PCR				71 387	
EBV nuclear antigen IgG			Negative	Positive
Viral capsid antigen IgM			Positive	Negative
Viral capsid antigen IgG			Positive	Positive

**Table 2:** Diagnostic criteria for haemophagocytic lymphohistiocytosis.

Diagnostic criterion	Case	Criteria score
1. A molecular diagnosis consistent with HLH	Positive	1
2. Fulfillment of five out of the eight criteria below:		
Fever (>100.4°*F*, >38°C)	Present	1
Splenomegaly	Absent	0
Cytopenias affecting at least two of three lineages in the peripheral blood		1
Haemoglobin <9 g/100 ml	Present	
Platelets <100 × 10^9^/L	Present	
Neutrophils <1 × 10^9^/L	Absent	
Hypertriglyceridemia and/or hypofibrinogenemia		1
Fasting triglyceride levels >3 mmol/liter	Present	
Fibrinogen level <1.5 g/liter	Present	
Ferritin ≥500 ng/ml	Present	1
Haemophagocytosis in bone marrow, central nervous system, spleen, or lymph nodes	Present	1
Low or absent natural killer cell activity	Not tested	
Soluble CD25 (soluble IL-2 receptor) > 2400 U/ml	Not tested	
**Total**		**6/8**

HLH-HaemophagocyticLymphohistiocytosis Criteria 1 and/or 2 must be present to make a diagnosis of HLH.
